# Serum and Histologic Eosinophilia as a Predictive Biomarker of Response to Mirikizumab in Ulcerative Colitis

**DOI:** 10.1093/ibd/izaf267

**Published:** 2025-11-24

**Authors:** Emily C L Wong, Parambir S Dulai, John K Marshall, Vipul Jairath, Walter Reinisch, Neeraj Narula

**Affiliations:** Division of Gastroenterology, Department of Medicine, McMaster University, Hamilton, ON, L8S 4K1, Canada; Farncombe Family Digestive Health Research Institute, McMaster University, Hamilton, ON, L8S 4K1, Canada; Division of Gastroenterology, Northwestern University, Chicago, IL, 60611, United States; Division of Gastroenterology, Department of Medicine, McMaster University, Hamilton, ON, L8S 4K1, Canada; Farncombe Family Digestive Health Research Institute, McMaster University, Hamilton, ON, L8S 4K1, Canada; Department of Medicine, Division of Gastroenterology, Western University, London, ON, N6A 3K7, Canada; Department of Internal Medicine III, Division of Gastroenterology and Hepatology, Medical University of Vienna, 1090, Vienna, Austria; Division of Gastroenterology, Department of Medicine, McMaster University, Hamilton, ON, L8S 4K1, Canada; Farncombe Family Digestive Health Research Institute, McMaster University, Hamilton, ON, L8S 4K1, Canada

**Keywords:** eosinophils, ulcerative colitis, mirikizumab

## Abstract

**Introduction:**

Eosinophils may contribute to ulcerative colitis (UC) pathogenesis through inflammation and epithelial injury. Mirikizumab, a selective IL-23 inhibitor, has shown efficacy in moderate-to-severe UC. However, the relationship between eosinophils and treatment outcomes with IL-23 inhibition remains unclear.

**Objective:**

This post-hoc analysis of the LUCENT-1 trial evaluated trends in serum and histologic eosinophils as biomarkers for early response to mirikizumab induction therapy in UC.

**Design:**

Data from LUCENT-1 (NCT03518086) were analyzed to assess serum eosinophil counts and histologic eosinophil presence at baseline and week 12. Clinical response, remission, and endoscopic improvement were defined by modified Mayo score criteria. Logistic regression models, adjusted for factors including steroid use, prior biologic failure, disease severity, and albumin levels, assessed associations between baseline eosinophil levels and treatment outcomes.

**Results:**

Among 928 participants, 66.6% achieved clinical response. Responders had higher baseline serum eosinophils (0.23 × 10^9^/L vs 0.19 × 10^9^/L, *P*  = .001) and showed greater reductions at week 12. Elevated baseline eosinophils (≥0.57 × 10^9^/L) were associated with higher rates of clinical response (80% vs 65.7%, aOR: 2.31, *P* = .013), remission (33.3% vs 25.1%, aOR: 1.91, *P* = .044), and endoscopic improvement (46.7% vs 33.8%, aOR: 2.33, *P*  = .003). Reductions in histologic eosinophils were also linked to improved outcomes, especially in those with moderate/marked baseline presence.

**Conclusions and Relevance:**

Baseline eosinophil levels may predict response to mirikizumab and guide early treatment decisions. These findings support a potential role for eosinophils as biomarkers in UC management.

Key Messages **What is already known?**

- Eosinophils are involved in the inflammatory process of ulcerative colitis (UC), and elevated counts have been associated with disease activity.
- Mirikizumab, an IL-23 inhibitor, has been shown to be effective in treating moderate-to-severe UC.
- Prior research with ustekinumab (an IL-12/23 inhibitor) has linked reductions in eosinophil levels to clinical response in UC and Crohn’s disease.
- However, the relationship between eosinophil levels (both serum and histologic) and treatment outcomes specifically with IL-23 inhibition had not been clearly defined.

**What is new here?**

- Higher baseline serum eosinophil levels (≥0.57 × 10^9^/L) were significantly associated with:
  - Greater clinical response (80% vs 65.7%, aOR: 2.31)
  - Clinical remission (33.3% vs 25.1%, aOR: 1.81)
  - Endoscopic improvement (46.7% vs 33.8%, aOR: 2.33)
- Declines in eosinophil levels during induction therapy correlated with better outcomes.
- Patients who experienced a rise in eosinophils during treatment were less likely to respond (aOR: 0.54).
- Histologic reductions in eosinophils by week 12 were also predictive of clinical and endoscopic improvements, especially among patients with moderate or marked eosinophil presence at baseline.
- These findings were consistent even without corticosteroid use, strengthening the case for eosinophils as independent biomarkers.

**How can this study help patient care?**

- Eosinophil monitoring could serve as an early biomarker of treatment response to mirikizumab in UC.
- Baseline eosinophil levels may help identify patients who are more likely to benefit from IL-23-targeted therapy.
- Failure to reduce eosinophils during induction could signal the need to consider alternative treatment strategies earlier.
- The study supports the idea that UC may have eosinophil-rich endotypes, which could eventually guide personalized therapy.
- Routine blood and biopsy eosinophil assessment could enhance therapeutic decision-making, reduce exposure to ineffective therapies, and improve long-term outcomes in UC.

## Introduction

Ulcerative colitis (UC) is a type of inflammatory bowel disease (IBD) characterized by chronic inflammation of the gastrointestinal tract. While the exact etiology of UC remains unclear, its pathogenesis likely involves several factors including genetic predisposition, environmental triggers, and dysregulated immune responses. Eosinophils are involved in immune and inflammatory responses with a growing body of evidence to suggest its role in the pathophysiology of autoimmune conditions such as UC.^1^

Normally, eosinophils reside within the lamina propria of the gastrointestinal tract, where they contribute to tissue homeostasis. However, in inflammatory states such as UC, eosinophils are recruited to intestinal tissues by chemoattractants, resulting in increased activation and degranulation.^2^^,^^3^ Activated eosinophils release inflammatory mediators, including major basic protein, eosinophil cationic protein, eosinophil peroxidase, and cytokines such as IL-5 and IL-13, which contribute to epithelial damage and amplify local inflammation. Elevated eosinophil counts in peripheral blood and intestinal tissue have been observed in patients with active UC, correlating with disease severity and inflammatory burden.^4^

Given the potential role of eosinophils in IBD pathogenesis, there is an increasing interest in assessing eosinophils as a biomarker of response to biologic therapies. Specifically, identifying markers of early response to biologics is important as it allows for timely optimization of treatment strategies, minimizing exposure to ineffective therapies and improving patient outcomes. Biologic therapies targeting key inflammatory pathways, such as interleukin (IL)-12, IL-23, and tumor necrosis factor (TNF), are used to treat moderate-severely active UC. IL-23 activation plays a critical role in UC by promoting the differentiation and expansion of pathogenic Th17 cells, which produce pro-inflammatory cytokines, contributing to sustained intestinal inflammation and epithelial damage. Eosinophil reduction was identified as an early marker of response to ustekinumab, a monoclonal antibody targeting IL-12 and IL-23, but not adalimumab, a TNF-alpha inhibitor, in both UC and CD.^5^ Mirikizumab is a monoclonal antibody selectively targeting IL-23, which has demonstrated efficacy in inducing clinical, endoscopic, and histologic remission in patients with moderate-to-severe UC. However, the relationship between eosinophils and treatment outcomes with IL-23 inhibitors remains largely unexplored. Understanding this relationship may improve early identification of patients with UC who are more likely to benefit from IL-23 inhibition. This study evaluates the trends in serum and histologic eosinophil levels as biomarkers of response to induction therapy with mirikizumab in UC.

## Methods

### Study Design

This was a post-hoc analysis of individual participant-level data from the LUCENT-1 clinical trial (ClinicalTrials.gov identifier: NCT03518086) which evaluated the safety and efficacy of mirikizumab compared to placebo for the treatment of moderate-severe UC. Data were obtained by permission from Eli Lilly and accessed through Vivli Inc. (protocol #00010341).^6^ Briefly, LUCENT-1 was an induction study where eligible participants were randomly assigned to receive 300 mg of mirikizumab or placebo intravenously every 4 weeks and were assessed for clinical remission at week 12. Eligible participants included those with modified Mayo score ≥ 4 with a Mayo endoscopic subscore (MES) ≥ 2 and prior treatment failure to steroids, immunomodulators, biologics, or janus kinase inhibitors. Concomitant use of oral 5-ASA, glucocorticoids, or immunomodulators was permitted during the induction trial. Endoscopies were performed at baseline and at week 12. Histologic assessments were performed using the Geboes Score at baseline at week 12, with eosinophil presence categorized as no increase, mild increase, moderate increase, or marked increase.^7^ All endoscopic assessments were centrally read.

### Outcomes

The primary outcome of this analysis was to assess the relationship of serum and histologic eosinophil levels with clinical response (decrease of ≥2 points in the modified Mayo score, with a decrease of ≥30% from baseline, plus a decrease from baseline of ≥1 point in rectal bleeding subscore [RBS] or RBS of 0 or 1). Secondary outcomes included clinical remission (stool frequency [SF] of 0 or SF of 1 with a decrease ≥1 point from baseline, RBS of 0, and MES < 2) and endoscopic improvement (MES < 2).

### Statistical Analysis

Baseline characteristics of the study population were summarized using descriptive statistics. Dichotomous variables were presented as proportions or percentages, while continuous variables were reported as means with standard deviations (SDs) or medians with interquartile ranges (IQRs). Categorical variables were compared using Chi-squared tests. Eosinophil counts were compared between responders and non-responders at each time point (baseline, week 4, 8, and 12) using t-tests to assess statistical significance. Changes in eosinophil counts from baseline to week 12 were analyzed both as absolute differences and percentage changes. For histologic analysis, participants were grouped based on baseline eosinophil presence as no increase/mild vs moderate/marked, as assessed based on the Geboes score. Sensitivity analyses were conducted separately for patients using baseline corticosteroids and those not. Logistic regression models were used to calculate odds ratios (ORs) and 95% confidence intervals (CIs) for achieving outcomes based on abnormally elevated baseline eosinophil counts. Trends and relationships identified through logistic regression were illustrated with lines of best fit, and least squares means were used to evaluate the average percent change for each comparison. A *P*-value < .05 was used to determine statistical significance.

## Results

### Demographics

The baseline characteristics of the study population are provided in Table 1. A total of 928 participants with moderate-severe UC who were treated with mirikizumab during induction with available data on serum and histologic eosinophil levels were included, of which 618 (66.6%) were clinical responders while 310 (33.4%) were non-responders. The mean age of participants was 42.8 years (SD: 13.8) and 38.3% of participants were female. The mean disease duration was 7.2 years (SD: 6.7). At baseline, 66.5% of participants had severe endoscopic disease while 33.5% had moderate disease. Corticosteroid use was reported in 15.7% of participants, and 17.7% were using immunomodulators. Mean baseline serum eosinophil counts were 0.22 × 10^9^/L (SD: 0.20), while the mean white blood cell, neutrophil, monocyte, and lymphocyte counts were 7.99, 5.44, 0.45, and 1.81 × 10^9^/L, respectively. No increase or mild increase in eosinophils was noted in histologic assessment among 84.0% of participants, while moderate or marked eosinophil increases were observed in 16.0%. Findings were similar across clinical responders and non-responders.

**Table 1.** Baseline characteristics of the study population.

Variable	Overall (N = 928)	Clinical responder (n = 618)	Clinical non-responder (n = 310)
**Age, mean (SD)**	42.8 (13.8)	42.3 (13.7)	43.9 (14.2)
**Female, n (%)**	355 (38.3)	251 (40.6)	104 (33.6)
**Disease duration in years, mean (SD)**	7.2 (6.7)	7.1 (6.4)	7.2 (7.1)
**Mayo endoscopic subscore, n (%)**			
** Moderate**	311 (33.5)	219 (35.4)	92 (29.7)
** Severe**	617 (66.5)	399 (64.6)	218 (70.3)
**Corticosteroid use at baseline, n (%) **	146 (15.7)	94 (15.2)	52 (16.8)
**Immunomodulator use at baseline, n (%) **	164 (17.7)	97 (15.7)	67 (21.6)
**Baseline eosinophil count (×10^9^/L), mean (SD)**	0.22 (0.2)	0.23 (0.2)	0.19 (0.2)
**Baseline white blood cell count (×10^9^/L), mean (SD)**	7.99 (2.9)	7.96 (2.9)	8.05 (2.9)
**Baseline neutrophil count (×10^9^/L), mean (SD)**	5.44 (2.5)	5.37 (2.5)	5.59 (2.6)
**Baseline monocyte count (×10^9^/L), mean (SD)**	0.45 (0.2)	0.46 (0.21)	0.45 (0.2)
**Baseline lymphocyte count (×10^9^/L), mean (SD)**	1.81 (0.8)	1.83 (0.84)	1.76 (0.7)
**Baseline histologic eosinophil presence, n (%)**			
** No increase**	407 (43.9)	259 (41.9)	148 (47.7)
** Mild increase**	372 (40.1)	269 (43.5)	103 (33.2)
** Moderate increase**	127 (13.7)	81 (13.1)	46 (14.8)
** Marked increase**	22 (2.4)	9 (1.5)	13 (4.2)

### Absolute Blood Eosinophil Count Trends During Mirikizumab Induction Therapy

The trends in absolute blood eosinophil counts during the 12-week induction phase with mirikizumab are summarized in Table 2. At baseline, clinical responders at week 12 had a significantly higher mean serum eosinophil count compared to non-responders (0.23 × 10^9^/L vs 0.19 × 10^9^/L, *P* = .001). However, no significant differences at baseline were observed between week 12 remitters and non-remitters (0.23 × 10^9^/L vs 0.21 × 10^9^/L, *P* = .363) or between those with and without week 12 endoscopic improvement (0.21 × 10^9^/L for both groups, *P* = .343). At week 4, eosinophil counts decreased across all groups, although there were no significant differences between responders and non-responders (0.20 × 10^9^/L vs 0.18 × 10^9^/L, *P* = .232), remitters and non-remitters (0.18 × 10^9^/L vs 0.20 × 10^9^/L, *P* = .124), or those achieving endoscopic improvement compared to those who did not (0.19 × 10^9^/L for both groups, *P* = .678). At week 8, eosinophil counts were significantly lower in remitters compared to non-remitters (0.15 × 10^9^/L vs 0.18 × 10^9^/L, *P* = .014) and in patients with endoscopic improvement compared to those without (0.16 × 10^9^/L vs 0.18 × 10^9^/L, *P* = .033). Responders had a numerically lower mean eosinophil count compared to non-responders at week 8, although this was not significant (0.17 × 10^9^/L vs 0.19 × 10^9^/L, *P* = .056). By week 12, eosinophil counts were further reduced. Significant differences were observed in remitters compared to non-remitters (0.13 × 10^9^/L vs 0.16 × 10^9^/L, *P* = .010) and in those achieving endoscopic improvement compared to those who did not (0.14 × 10^9^/L vs 0.16 × 10^9^/L, *P* = .027). While counts were lower in responders compared to non-responders (0.15 × 10^9^/L vs 0.16 × 10^9^/L), this difference did not reach statistical significance (*P* = .227).

**Table 2.** Absolute blood eosinophil count trends in patients treated with mirikizumab for ulcerative colitis during induction (n = 928).

Mean eosinophil count, ×10^9^/L (SD)	Week 12 clinical response			Week 12 clinical remission			Week 12 endoscopic improvement		
	Non-responders (n = 310)	Responders (n = 618)	*P*	Non-remitters (n = 690)	Remitters (n = 238)	*P*	Not achieved (n = 607)	Achieved (n = 321)	*P*
**Week 0**	0.19 (0.16)	0.23 (0.22)	.001	0.21 (0.20)	0.23 (0.20)	.363	0.21 (0.23)	0.21 (0.19)	.343
**Week 4**	0.18 (0.15)	0.20 (0.17)	.232	0.20 (0.17)	0.18 (0.15)	.124	0.19 (0.15)	0.19 (0.17)	.678
**Week 8**	0.19 (0.17)	0.17 (0.14)	.056	0.18 (0.16)	0.15 (0.12)	.014	0.18 (0.17)	0.16 (0.12)	.033
**Week 12**	0.16 (0.14)	0.15 (0.14)	.227	0.16 (0.15)	0.13 (0.10)	.010	0.16 (0.15)	0.14 (0.11)	.027
**Week 8 absolute delta from baseline**	-0.01 (0.16)	-0.07 (0.19)	<.001	-0.03 (0.18)	-0.07 (0.18)	.005	-0.03 (0.17)	-0.08 (0.20)	<.001
**Week 8 percent delta from baseline**	-1.36% (9.43)	-32.55% (14.30)	<.001	-7.22% (11.43)	-15.36% (11.26)	.076	-14.29% (12.13)	-38.10% (9.78)	.009
**Week 12 absolute delta from baseline**	-0.02 (0.15)	-0.08 (0.19)	<.001	-0.05 (0.18)	-0.10 (0.18)	.002	-0.05 (0.20)	-0.08 (0.17)	<.001
**Week 12 percent delta from baseline**	-10.53% (15.16)	-34.78% (9.18)	<.001	-23.81% (11.92)	-43.48% (9.93)	.032	-23.81% (12.11)	-38.1% (10.10)	.017

Clinical response: decrease of ≥2 points in the modified Mayo score, with a decrease of ≥30% from baseline, plus a decrease from baseline of ≥1 point in RBS or RBS of 0 or 1.

Clinical remission: SF of 0 or SF of 1 with a decrease ≥1 point from baseline, RBS of 0, and MES of 0 or 1.

Endoscopic improvement: Mayo endoscopic subscore < 2.

**Figure 1.**
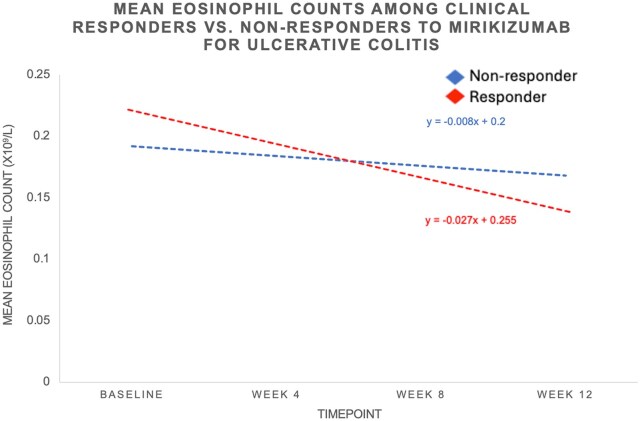
Trends in absolute blood eosinophil counts in patients treated with mirikizumab for ulcerative colitis during induction by week 12 clinical response.

**Figure 2.**
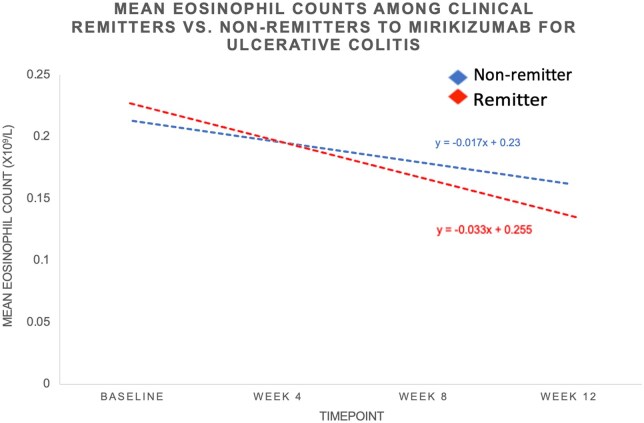
Trends in absolute blood eosinophil counts in patients treated with mirikizumab for ulcerative colitis during induction by week 12 clinical remission.

**Figure 3.**
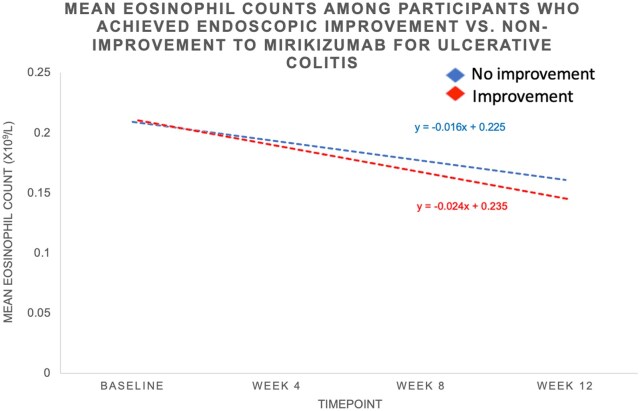
Trends in absolute blood eosinophil counts in patients treated with mirikizumab for ulcerative colitis during induction by week 12 endoscopic improvement.

The absolute and percentage reductions in eosinophil counts from baseline to week 8 and from baseline to week 12 were also assessed. Responders had a significantly greater absolute reduction in eosinophil counts compared to non-responders at week 8 (–0.07 × 10^9^/L vs –0.03 × 10^9^/L, *P* < .001) and week 12 (−0.08 × 10^9^/L vs −0.02 × 10^9^/L, *P* < .001). Similarly, remitters had a larger absolute decrease at week 8 (–0.07 × 10^9^/L vs –0.03 × 10^9^/L, *P* = .005) and week 12 (−0.10 × 10^9^/L vs −0.05 × 10^9^/L, *P* = .002), as did those achieving endoscopic improvement at week 8 (–0.08 × 10^9^/L vs -0.03 × 10^9^/L, *P* < .001) and week 12 (−0.08 × 10^9^/L vs −0.05 × 10^9^/L, *P* < .001). Percentage reductions were also greater in responders at week 8 (–32.55% vs –1.36%, *P* < .001) and week 12 (−34.78% vs −10.53%, *P* < .001), remitters at week 12 (−43.48% vs −23.81%, *P* = .032), and those with endoscopic improvement at week 8 (–38.10% vs –14.29%, *P* = .009) and week 12 (−38.10% vs −23.81%, *P* = .017). The least square means difference at week 12 for the outcome of clinical response was 21% among responders and 12% among non-responders. For clinical remission, the least square means difference was 25% among remitters compared to 9% among non-remitters. For endoscopic improvement, the least square means difference was 20% among those with improvement compared to 10% among those without improvement. Figures 1-3 illustrate the trends in absolute blood eosinophil counts over 12 weeks across clinical response, remission, and endoscopic improvement groups. In all 3 outcomes, responders exhibited a steeper decline in serum eosinophil levels compared to non-responders. The greatest absolute reduction was observed among those achieving clinical remission (Figure 2), followed by those with clinical response (Figure 1) and endoscopic improvement (Figure 3). Table S1 demonstrates trends in eosinophil counts among patients without concomitant steroid use. Similar findings were observed across all outcomes assessed.

### Baseline Serum Eosinophil Levels and Post-induction Response

Table 3 outlines the association between baseline serum eosinophil levels and post-induction outcomes. Participants with abnormally elevated baseline eosinophil counts (≥0.57 × 10^9^/L) were significantly more likely to achieve clinical response at week 12 compared to those with lower eosinophil counts after adjustment for key covariates (concomitant steroid use, prior biologic failure, severe Modified Mayo score at baseline, and albumin level at baseline) (80.0% vs 65.7%, aOR: 2.31 [95% CI: 1.19-4.50], *P* = .013). Similarly, clinical remission was significantly more likely among participants with elevated baseline eosinophils (33.3% vs 25.1%, aOR: 1.81 [95% CI: 1.02-3.23], *P* = .044). Endoscopic improvement was also significantly more likely among participants with elevated baseline eosinophils (46.7% vs 33.8%, aOR: 2.33 [95% CI: 1.33-4.09], *P* = .003). Similar findings were observed among patients without concomitant steroid use for clinical response (80% vs 66.1%, aOR: 2.37 [95% CI: 1.15-4.91], *P* = .020), clinical remission (32.0% vs 25.7%, aOR: 1.63 [95% CI: 0.86-3.08, *P* = .137], and endoscopic improvement (52.0% vs 34.3%, aOR: 2.94 [95% CI: 1.59-5.46], *P* = .001) among those with elevated eosinophils at baseline compared to those with lower levels (Table S2).

**Table 3.** Comparison of post-induction (week 12) response rates stratified by baseline absolute serum eosinophil counts.

	Elevated eosinophils at baseline (ULN of 0.57)′	Not elevated eosinophils at baseline	Adjusted OR (95% CI)^a^	*P*
**Week 12 clinical response, n (%)**	48/60 (80.0)	570/868 (65.7)	2.31 (1.19-4.50)	.013
**Week 12 clinical remission, n (%)**	20/60 (33.3)	218/868 (25.1)	1.81 (1.02-3.23)	.044
**Week 12 endoscopic improvement, n (%)**	28/60 (46.7)	293/868 (33.8)	2.33 (1.33-4.09)	.003

Clinical response: decrease of ≥2 points in the modified Mayo score, with a decrease of ≥30% from baseline, plus a decrease from baseline of ≥1 point in RBS or RBS of 0 or 1.

Clinical remission: SF of 0 or SF of 1 with a decrease ≥1 point from baseline, RBS of 0, and MES of 0 or 1.

Endoscopic improvement: Mayo endoscopic subscore < 2.

a Adjusted for concomitant steroid use, prior biologic failure, severe Modified Mayo score at baseline and albumin level at baseline.

Table 4 demonstrates the impact of a rise in serum eosinophil counts from baseline to week 12 on treatment outcomes. Patients who experienced any rise in eosinophils were significantly less likely to achieve clinical response at week 12 compared to those who did not (61.8% vs 74.9%, aOR: 0.54 [95% CI: 0.40–0.73], *P* < .001). Similarly, rates of clinical remission (22.5% vs 31.0%, aOR: 0.65 [95% CI: 0.48–0.88], *P* = .005) and endoscopic improvement (31.1% vs 40.6%, aOR: 0.63 [95% CI: 0.46–0.82], *P* = .002) were significantly lower in patients with a rise in eosinophil counts. Table S3 further stratifies response rates based on the magnitude of eosinophil increase, demonstrating that patients with a >50% rise in eosinophils from baseline to week 12 had significantly lower rates of clinical response (61.4% vs 70.1%, aOR: 0.67 [95% CI: 0.49–0.9], *P* = .008), clinical remission (20.3% vs 29.2%, aOR: 0.63 [95% CI: 0.46–0.86], *P* = .004), and endoscopic improvement (29.5% vs 38.0%, aOR: 0.69 [95% CI: 0.52–0.92], *P* = .011) compared to those with a ≤ 50% rise.

**Table 4.** Comparison of post-induction (week 12) response rates stratified by rise in serum eosinophil counts.

	Any rise in eosinophils from baseline to week 12^a^	No rise in serum eosinophils from baseline to week 12^a^	Adjusted OR (95% CI)^b^	*P*-value
**Week 12 clinical response, n (%)**	362/586 (61.8)	256/342 (74.9)	0.54 (0.40-0.73)	<.001
**Week 12 clinical remission, n (%)**	132/586 (22.5)	106/342 (31.0)	0.65 (0.48-0.88)	.005
**Week 12 endoscopic improvement, n (%)**	182/586 (31.1)	139/342 (40.6)	0.63 (0.46-0.82)	.002

Clinical response: decrease of ≥2 points in the modified Mayo score, with a decrease of ≥30% from baseline, plus a decrease from baseline of ≥1 point in RBS or RBS of 0 or 1.

Clinical remission: SF of 0 or SF of 1 with a decrease ≥1 point from baseline, RBS of 0, and MES of 0 or 1.

Endoscopic improvement: Mayo endoscopic subscore < 2.

a Eosinophils assessed at week 4, 8, and 12.

b Adjusted for concomitant steroid use, prior biologic failure, severe Modified Mayo score at baseline and albumin level at baseline.

### Histologic Eosinophil Presence and Outcomes

Table 5 evaluates histologic eosinophil presence at baseline and week 12 in relation to clinical and endoscopic outcomes. Among patients with moderate or marked histologic eosinophil increase at baseline, a significantly greater proportion of week 12 responders had a reduction in eosinophils to none/mild compared to non-responders (62.2% vs 50.9%, *P* < .001). Similarly, a greater proportion of those who achieved week 12 endoscopic improvement experienced a reduction in histologic eosinophils compared to those without endoscopic improvement (68.1% vs 52.9%, *P* < .001). Sensitivity analyses further explored these findings, comparing moderate/marked eosinophil presence at baseline to no increase/mild levels. Notably, in those with moderate/marked histologic eosinophils at baseline, the odds of achieving clinical remission were significantly higher for patients treated with mirikizumab versus placebo after adjusting for concomitant steroid use, prior biologic failure, severe Modified Mayo score at baseline and albumin level at baseline (adjusted OR [aOR]: 3.32 [95% CI: 1.20-9.22], *P* = .021), which was markedly higher than the odds of response in mirikizumab versus placebo treated patients who had no increase or mild increase in histologic eosinophils at baseline (aOR: 1.73 [95% CI: 1.19-2.52], *P* = .004). Similar findings were observed when analyses were restricted to those who were not using steroids concomitantly in the moderate/marked subgroup (aOR: 3.80 [95% CI: 1.23-11.67], *P* = .020) compared to the no increase/mild increase subgroup (aOR: 1.67 [95% CI: 1.11-2.52], *P* = .014). The relationship between serum and histologic eosinophils was also evaluated based on baseline serum eosinophil levels. A similar proportion of participants with abnormally elevated baseline serum eosinophil counts had moderate/marked increases in histologic eosinophils compared to those without elevated serum eosinophils at baseline (11/60 [18.3%] vs. 138/868 [15.9%], *P* = .619).

**Table 5.** Comparison of post-induction (week 12) response on histologic eosinophil presence in patients treated with mirikizumab for ulcerative colitis during induction (n = 928).

Overall (*P* < .001)		
**Baseline**	Week 12	
	No increase/Mild	Moderate/Marked
**No increase/Mild**	666/779 (85.5)	113/779 (14.5)
**Moderate/Marked**	86/149 (57.7)	63/149 (42.3)
**Patients who achieved clinical response at week 12 (*P* < .001)**		
**Baseline**	Week 12	
	No increase/Mild	Moderate/Marked
**No increase/Mild**	447/528 (84.7)	81/528 (15.3)
**Moderate/Marked**	56/90 (62.2)	34/90 (37.8)
**Patients who did not achieve clinical response at week 12 (*P* < .001)**		
**Baseline**	Week 12	
	No increase/Mild	Moderate/Marked
**No increase/Mild**	219/251 (87.3)	32/251 (12.8)
**Moderate/Marked**	30/59 (50.9)	29/59 (49.2)
**Patients who achieved endoscopic improvement at week 12 (*P* < .001)**		
**Baseline**	Week 12	
	No increase/Mild	Moderate/Marked
**No increase/Mild**	250/274 (91.2)	24/274 (8.8)
**Moderate/Marked**	32/47 (68.1)	15/47 (31.9)
**Patients who did not achieve endoscopic improvement at week 12 (*P* < .001)**		
**Baseline**	Week 12	
	No increase/Mild	Moderate/Marked
**No increase/Mild**	416/505 (82.4)	89/505 (17.6)
**Moderate/Marked**	54/102 (52.9)	48/102 (47.1)

Table S4 provides a sensitivity analysis of histologic eosinophil presence at baseline and week 12 in relation to outcomes among patients without concomitant steroid use. Again, among those with moderate/marked increase in eosinophils at baseline, responders were significantly more likely to achieve reduction in eosinophils (no/mild increase) by week 12 (61.7% vs 50.9%, *P* < .001) compared to non-responders. Similarly, those who achieved endoscopic improvement with moderate/marked increase in eosinophils at baseline were significantly more likely to achieve reduction (64.3% vs 54.2%, *P* < .001). These findings were consistent when individual categories of eosinophils were assessed (Table S5).

## Discussion

There is growing interest in evaluating serum and histologic biomarkers not only for assessing disease activity but also for their potential to predict treatment response.^8^

Previous post-hoc analyses of clinical trial data have shown that a decline in blood eosinophil counts following induction therapy with ustekinumab is associated with a greater treatment response in both CD and UC.^5^ Similarly, in this study, we found that higher baseline eosinophil levels were associated with an increased likelihood of achieving clinical response and endoscopic improvement at week 12 to mirikizumab. Moreover, responders demonstrated a significantly greater absolute and percentage reduction in eosinophil counts from baseline to week 12 compared to non-responders, further supporting the role of eosinophils as a potential biomarker of treatment response with mirikizumab in patients with UC.

The observed association between eosinophil levels and response to mirikizumab may be explained by the mechanistic role of IL-23 in eosinophil regulation. IL-23 has been implicated in the recruitment and activation of eosinophils, contributing to the inflammatory cascade in UC, particularly in the context of Th17 cell differentiation and activation.^9^ The importance of IL-23 in this context is further emphasized when compared to IL-12. While both IL-12 and IL-23 share the common p40 subunit, their biological effects have some differences worth noting.^10^ IL-12 is primarily involved in promoting Th1 immune responses, driving the production of IFN-γ, which is important in defense against intracellular pathogens.^11^ However, IL-23 has a more specific role in driving Th17 responses, particularly in the context of autoimmune and chronic inflammatory diseases such as UC. Activation of the Th17 pathway leads to production of IL-17, which further amplifies the recruitment and activation of inflammatory cells, including eosinophils.^12^ As IL-23 plays a more direct role in the regulation of eosinophils compared to IL-12, therapies that selectively target IL-23 such as mirikizumab offer a more specific means of modulating the inflammatory processes that involve eosinophils and Th17 cells in UC, potentially providing greater therapeutic benefit. By selectively targeting IL-23, mirikizumab may reduce eosinophil activation and tissue infiltration, resulting in decreased inflammation and improved clinical outcomes. This is consistent with prior studies evaluating IL-12/23 blockade with ustekinumab, where a similar decline in eosinophil counts was identified as an early marker of response.^5^ In our analysis, we observed a progressive decline in serum eosinophil counts among responders (Figures 1-3). Notably, patients achieving clinical remission showed the most pronounced eosinophil reduction, suggesting that deeper disease control may correlate with greater suppression of eosinophilic activity. Furthermore, we observed that failure to suppress serum eosinophil levels during induction therapy may be associated with a reduced likelihood of achieving key clinical and endoscopic endpoints.

Given these findings, monitoring eosinophil levels during induction therapy may help clinicians identify patients who are more likely to benefit from mirikizumab and optimize treatment strategies accordingly. In contrast, anti-TNF therapies such as adalimumab do not act upstream on IL-23 and may have a less pronounced effect on eosinophil-mediated inflammation.^13^^,^^14^ Our analyses also demonstrated that at baseline, participants with abnormally elevated serum eosinophil counts (≥0.57 × 10^9^/L) were significantly more likely to achieve clinical response and endoscopic improvement at week 12 compared to those with lower eosinophil levels at baseline, which may serve as an additional marker for identifying patients who are more likely to respond to mirikizumab treatment.

Our findings also highlight the predictive value of histologic eosinophils in identifying treatment responders. Among participants with moderate or marked eosinophil presence at baseline, those achieving clinical and endoscopic improvement were significantly more likely to experience a reduction in eosinophil levels by week 12. The OR for achieving clinical remission was almost double for patients with moderate/marked histologic eosinophils at baseline when treated with mirikizumab versus placebo compared to patients who had no/mild increase in histologic eosinophils at baseline. This suggests that patients with higher initial eosinophil counts may be more responsive to IL-23-targeted therapy such as mirikizumab. Notably, these associations remained consistent in sensitivity analyses excluding patients on concomitant corticosteroids. We evaluated the association between serum and histologic eosinophils and found no significant difference in the proportion of participants with a moderate/marked increase in histologic eosinophils at baseline between those with abnormally elevated serum eosinophils and those without. Notably, most clinical responders did not have elevated eosinophils in either serum or histology at baseline. This suggests that normal serum or histologic eosinophil counts should not be a barrier to initiating anti-IL-23 treatment. However, additional consideration for this therapy may be warranted among patients with elevated baseline serum or histologic eosinophils. Histologic assessment of eosinophils provides a more localized and direct measure of eosinophil infiltration within the intestinal mucosa, offering insights into the inflammatory processes that may not be fully captured by peripheral blood eosinophil counts alone. This is particularly important in UC, where inflammation is often confined to the mucosal layer of the colon and rectum. An advantage of histologic assessment is that it can directly correlate the level of eosinophil infiltration with disease severity, providing a more specific picture of underlying disease processes.

The implications of these findings extend to treatment approaches in UC. High baseline eosinophil counts could help identify patients more likely to respond to IL-23 inhibition, while failure to achieve eosinophil reduction may warrant the consideration of other treatment options. Monitoring eosinophil levels during induction therapy with mirikizumab may provide clinicians with an early indicator of treatment efficacy. Furthermore, in lights of similar observations with ustekinumab, our findings also raise the question as to whether an eosinophil-rich UC endotype exists, which has important clinical implications.^15^ Theoretically, UC patients with eosinophil-driven inflammation may benefit more from eosinophil-targeted therapies, although additional research is warranted to assess this.

Our study has several strengths, including use of individual participant-level clinical trial data with centrally read endoscopy and histology. However, several limitations should be acknowledged. First, this study was a post-hoc analysis of clinical trial data, and external validation in real-world cohorts is necessary to confirm the utility of eosinophils as a biomarker of response. Second, while we adjusted for key covariates, residual confounding cannot be ruled out. As this was a post-hoc analysis, histologic assessment of eosinophils was restricted to the categorical framework of the Geboes score, which may not capture all the nuanced details of eosinophil infiltration. Moreover, there are no standardized definitions for strata of eosinophilia using the Geboes score. More granular or refined methods of eosinophil evaluation could provide further insights into the inflammatory process. Lastly, while we assessed both absolute and percentage reductions in eosinophil counts, the specific threshold of reduction that would be clinically meaningful or most predictive of treatment success remains uncertain. Future studies with larger datasets should investigate the role of eosinophils in predicting long-term treatment outcomes, including sustained remission and histologic healing.

In conclusion, our study demonstrates that both serum and histologic eosinophil levels may serve as valuable biomarkers for responders to mirikizumab in UC. The significant reductions in eosinophil counts observed among responders suggest that IL-23 inhibition may effectively target eosinophil-mediated inflammation. These findings support ongoing research into eosinophil-based biomarkers and therapeutic strategies to improve IBD management.

## Supplementary Material

izaf267_Supplementary_Data

## Data Availability

This publication (Vivli protocol #00010341) is based on research using data from data contributors Eli Lilly that has been made available through Vivli, Inc. Vivli has not contributed to or approved, and Vivli and Eli Lilly are not in any way responsible for, the contents of this publication.
