# Supplementary material for: Serum and Histologic Eosinophilia as a Predictive Biomarker of Response to Mirikizumab in Ulcerative Colitis
Source: Inflamm Bowel Dis. 2025 Nov 24;32(4):634–42. doi: 10.1093/ibd/izaf267 (PMC13046049; doi:10.1093/ibd/izaf267)
Supplement: izaf267_Supplementary_Data [file izaf267_supplementary_data.zip › Supplementary_appendix .docx]

Supplementary Table 1 – Absolute blood eosinophil count trends in patients without concomitant steroid use treated with mirikizumab for ulcerative colitis during induction (n=782)

| Mean eosinophil count, x10^9^/L (SD) | Week 12 Clinical Response | | | Week 12 Clinical Remission | | | Week 12 Endoscopic Improvement | | |
| --- | --- | --- | --- | --- | --- | --- | --- | --- | --- |
|  | Non-responders (n=378) | Responders (n=404) | p-value | Not-achieved (n=644) | Achieved (n=138) | p-value | Not achieved (n=505) | Achieved (n=277) | p-value |
| Week 0 | 0.19 (0.15) | 0.24 (0.22) | 0.002 | 0.22 (0.20) | 0.23 (0.20) | 0.580 | 0.21 (0.18) | 0.25 (0.23) | 0.015 |
| Week 4 | 0.19 (0.16) | 0.20 (0.18) | 0.208 | 0.20 (0.18) | 0.18 (0.15) | 0.148 | 0.20 (0.18) | 0.20 (0.16) | 0.842 |
| Week 8 | 0.19 (0.18) | 0.17 (0.15) | 0.047 | 0.18 (0.17) | 0.15 (0.12) | 0.028 | 0.18 (0.18) | 0.16 (0.12) | 0.071 |
| Week 12 | 0.17 (0.14) | 0.15 (0.14) | 0.146 | 0.16 (0.15) | 0.13 (0.10) | 0.014 | 0.16 (0.16) | 0.14 (0.11) | 0.055 |
| Week 12 absolute delta from baseline | -0.02 (0.14) | -0.09 (0.19) | <0.001 | -0.06 (0.18) | -0.10 (0.18) | 0.012 | -0.05 (0.17) | -0.11 (0.20) | <0.001 |
| Week 12 percent delta from baseline | -10.53% (13.41) | -37.5% (8.53) | <0.001 | -27.27% (10.78) | -43.48% (9.05) | 0.056 | -23.81% (10.53) | -44% (10.03) | 0.076 |

Clinical Response: decrease of ≥2 points in the modified Mayo score, with a decrease of ≥30% from baseline, plus a decrease from baseline of ≥1 point in RBS or RBS of 0 or 1

Clinical Remission: SF of 0 or SF of 1 with a decrease ≥1 point from baseline, RBS of 0, and MES of 0 or 1

Endoscopic improvement: Mayo endoscopic subscore < 2

Supplementary Table 2 – Comparison of post-induction (week 12) response rates among patients without concomitant steroid use stratified by baseline absolute serum eosinophil counts

|  | Elevated eosinophils at baseline (ULN of 0.57) | Not elevated eosinophils at baseline | Adjusted OR (95% CI)* | AUC (95% CI) | p-value |
| --- | --- | --- | --- | --- | --- |
| Week 12 Clinical Response, n (%) | 40/50 (80.0) | 484/732 (66.1) | 2.37 (1.15-4.91) | 0.53 (0.51-0.55) | 0.020 |
| Week 12 Clinical Remission, n (%) | 16/50 (32.0) | 188/732 (25.7) | 1.63 (0.86-3.08) | 0.52 (0.49-0.54) | 0.137 |
| Week 12 Endoscopic Improvement, n (%) | 26/50 (52.0) | 251/732 (34.3) | 2.94 (1.59-5.46) | 0.53 (0.51-0.55) | 0.001 |

Clinical Response: decrease of ≥2 points in the modified Mayo score, with a decrease of ≥30% from baseline, plus a decrease from baseline of ≥1 point in RBS or RBS of 0 or 1

Clinical Remission: SF of 0 or SF of 1 with a decrease ≥1 point from baseline, RBS of 0, and MES of 0 or 1

Endoscopic improvement: Mayo endoscopic subscore < 2

*Adjusted for prior biologic failure, severe Modified Mayo score at baseline and albumin level at baseline

Supplementary Table 3 – Comparison of post-induction (week 12) response rates stratified by rise in serum eosinophil counts

|  | Rise in eosinophils from baseline to week 12 > 50%** | ≤ 50% rise in serum eosinophils from baseline to week 12** | Adjusted OR (95% CI)* | AUC (95% CI) | p-value |
| --- | --- | --- | --- | --- | --- |
| Week 12 Clinical Response, n (%) | 227/370 (61.4) | 391/558 (70.1) | 0.67 (0.49-0.90) | 0.45 (0.42-0.48) | 0.008 |
| Week 12 Clinical Remission, n (%) | 75/370 (20.3) | 163/558 (29.2) | 0.63 (0.46-0.86) | 0.44 (0.41-0.48) | 0.004 |
| Week 12 Endoscopic Improvement, n (%) | 109/370 (29.5) | 212/558 (38.0) | 0.69 (0.52-0.92) | 0.45 (0.42-0.48) | 0.011 |

Clinical Response: decrease of ≥2 points in the modified Mayo score, with a decrease of ≥30% from baseline, plus a decrease from baseline of ≥1 point in RBS or RBS of 0 or 1

Clinical Remission: SF of 0 or SF of 1 with a decrease ≥1 point from baseline, RBS of 0, and MES of 0 or 1

Endoscopic improvement: Mayo endoscopic subscore < 2

*Adjusted for concomitant steroid use, prior biologic failure, severe Modified Mayo score at baseline and albumin level at baseline

**Eosinophils assessed at week 4, 8 and 12

Supplementary Table 4 – Comparison of post-induction (week 12) response on histologic eosinophil presence in patients treated with mirikizumab for ulcerative colitis during induction stratified by any eosinophils vs. none (n=928)

| **Overall (p<0.001)** | | |
| --- | --- | --- |
| Baseline | Week 12 | |
|  | No increase | Mild/Moderate / Marked |
| No increase (n=407) | 288 (70.8) | 119 (29.2) |
| Mild/Moderate / Marked (n=521) | 308 (59.1) | 213 (40.9) |
| **Patients who achieved clinical response at week 12 (p=0.003)** | | |
| Baseline | Week 12 | |
|  | No increase | Mild/Moderate / Marked |
| No increase (n=259) | 184 (71.0) | 75 (29.0) |
| Mild/Moderate / Marked (n=359) | 214 (59.6) | 145 (40.4) |
| **Patients who did not achieve clinical response at week 12 (p=0.025)** | | |
| Baseline | Week 12 | |
|  | No increase | Mild/Moderate / Marked |
| No increase (n=148) | 104 (70.3) | 44 (29.7) |
| Mild/Moderate / Marked (n=162) | 94 (58.0) | 68 (42.0) |
| **Patients who achieved endoscopic improvement at week 12 (p=0.002)** | | |
| Baseline | Week 12 | |
|  | No increase | Mild/Moderate / Marked |
| No increase (n=130) | 99 (76.2) | 31 (23.9) |
| Mild/Moderate / Marked (n=191) | 113 (59.2) | 78 (40.8) |
| **Patients who did not achieve endoscopic improvement at week 12 (p=0.020)** | | |
| Baseline | Week 12 | |
|  | No increase | Mild/Moderate / Marked |
| No increase (n=277) | 189 (68.2) | 88 (31.8) |
| Mild/Moderate / Marked (n=330) | 195 (59.1) | 135 (40.9) |

Supplementary Table 5 – Comparison of post-induction (week 12) response on histologic eosinophil presence in patients without concomitant steroid use treated with mirikizumab for ulcerative colitis during induction (n=782)

| **Overall (p<0.001)** | | |
| --- | --- | --- |
| Baseline | Week 12 | |
|  | No increase / Mild | Moderate / Marked |
| No increase / Mild | 548/644 (85.1) | 96/644 (14.9) |
| Moderate / Marked | 79/138 (57.3) | 59/138 (42.8) |
| **Patients who achieved clinical response at week 12 (p<0.001)** | | |
| Baseline | Week 12 | |
|  | No increase / Mild | Moderate / Marked |
| No increase / Mild | 373/443 (84.2) | 70/443 (15.8) |
| Moderate / Marked | 50/81 (61.7) | 31/81 (38.3) |
| **Patients who did not achieve clinical response at week 12 (p<0.001)** | | |
| Baseline | Week 12 | |
|  | No increase / Mild | Moderate / Marked |
| No increase / Mild | 175/201 (87.1) | 26/201 (12.9) |
| Moderate / Marked | 29/57 (50.9) | 28/57 (49.1) |
| **Patients achieved endoscopic improvement at week 12 (p<0.001)** | | |
| Baseline | Week 12 | |
|  | No increase / Mild | Moderate / Marked |
| No increase / Mild | 213/235 (90.6) | 22/235 (9.4) |
| Moderate / Marked | 27/42 (64.3) | 15/42 (35.7) |
| **Patients who did not achieve endoscopic improvement at week 12 (p<0.001)** | | |
| Baseline | Week 12 | |
|  | No increase / Mild | Moderate / Marked |
| No increase / Mild | 335/409 (81.9) | 74/409 (18.1) |
| Moderate / Marked | 52/96 (54.2) | 44/96 (45.8) |

Supplementary Table 6 – Comparison of post-induction (week 12) response on histologic eosinophil presence in patients treated with mirikizumab for ulcerative colitis during induction stratified by eosinophil category (n=928)

| **Overall (p<0.001)** | | | | |
| --- | --- | --- | --- | --- |
| Baseline | Week 12 | | | |
|  | No increase | Mild | Moderate | Marked |
| No increase (n=407) | 242 (59.5) | 119 (29.2) | 42 (10.3) | 4 (1.0) |
| Mild (n=372) | 140 (37.6) | 165 (44.4) | 58 (15.6) | 9 (2.4) |
| Moderate (n=127) | 35 (27.6) | 42 (33.1) | 39 (30.7) | 11 (8.7) |
| Marked (n=22) | 3 (13.6) | 6 (27.3) | 9 (40.9) | 4 (18.2) |
| **Week 12 clinical responders (p<0.001)** | | | | |
| Baseline | Week 12 | | | |
|  | No increase | Mild | Moderate | Marked |
| No increase (n=259) | 153 (59.1) | 75 (29.0) | 28 (10.8) | 3 (1.2) |
| Mild (n=269) | 104 (38.7) | 115 (42.8) | 43 (16.0) | 7 (2.6) |
| Moderate (n=81) | 24 (29.6) | 26 (32.1) | 24 (29.6) | 7 (8.6) |
| Marked (n=9) | 2 (22.2) | 4 (44.4) | 3 (33.3) | 0 |
| **Week 12 clinical non-responders (p<0.001)** | | | | |
| Baseline | Week 12 | | | |
|  | No increase | Mild | Moderate | Marked |
| No increase (n=148) | 89 (60.1) | 44 (29.7) | 14 (9.5) | 1 (0.7) |
| Mild (n=103) | 36 (35.0) | 50 (48.5) | 15 (14.6) | 2 (1.9) |
| Moderate (n=46) | 11 (23.9) | 16 (34.8) | 15 (32.6) | 4 (8.7) |
| Marked (n=13) | 1 (7.7) | 2 (15.4) | 6 (46.2) | 4 (30.8) |
